# Efficacy of subsequent treatments in patients with hormone-positive advanced breast cancer who had disease progression under CDK 4/6 inhibitor therapy

**DOI:** 10.1186/s12885-023-10609-8

**Published:** 2023-02-10

**Authors:** Cengiz Karacin, Berna Oksuzoglu, Ayşe Demirci, Merve Keskinkılıç, Naziyet Köse Baytemür, Funda Yılmaz, Oğuzhan Selvi, Dilek Erdem, Esin Avşar, Nail Paksoy, Necla Demir, Sema Sezgin Göksu, Sema Türker, Ertuğrul Bayram, Abdüssamet Çelebi, Hatice Yılmaz, Ömer Faruk Kuzu, Seda Kahraman, İvo Gökmen, Abdullah Sakin, Ali Alkan, Erdinç Nayır, Muzaffer Uğraklı, Ömer Acar, İsmail Ertürk, Hacer Demir, Ferit Aslan, Özlem Sönmez, Taner Korkmaz, Özde Melisa Celayir, İbrahim Karadağ, Erkan Kayıkçıoğlu, Teoman Şakalar, İlker Nihat Öktem, Tülay Eren, Enes Urul, Eda Eylemer Mocan, Ziya Kalkan, Nilgün Yıldırım, Yakup Ergün, Baran Akagündüz, Serdar Karakaya, Engin Kut, Fatih Teker, Burçin Çakan Demirel, Kubilay Karaboyun, Elvina Almuradova, Olçun Ümit Ünal, Abdilkerim Oyman, Deniz Işık, Kerem Okutur, Buğra Öztosun, Burcu Belen Gülbağcı, Mehmet Emin Kalender, Elif Şahin, Mustafa Seyyar, Özlem Özdemir, Fatih Selçukbiricik, Metin Kanıtez, İsa Dede, Mahmut Gümüş, Erhan Gökmen, Arzu Yaren, Serkan Menekşe, Senar Ebinç, Sercan Aksoy, Gökşen İnanç İmamoğlu, Mustafa Altınbaş, Bülent Çetin, Başak Oyan Uluç, Özlem Er, Nuri Karadurmuş, Atike Pınar Erdoğan, Mehmet Artaç, Özgür Tanrıverdi, İrfan Çiçin, Mehmet Ali Nahit Şendur, Esin Oktay, İbrahim Vedat Bayoğlu, Semra Paydaş, Adnan Aydıner, Derya Kıvrak Salim, Çağlayan Geredeli, Tuğba Yavuzşen, Mutlu Doğan, İlhan Hacıbekiroğlu

**Affiliations:** 1 Department of Medical Oncology, UHS Dr Abdurrahman Yurtaslan Ankara Oncology Training and Research Hospital, Ankara, Turkey; 2 Department of Medical Oncology, Sakarya University, Sakarya, Turkey; 3 Department of Medical Oncology, Dokuz Eylül University, İzmir, Turkey; 4 Department of Medical Oncology, Memorial Hospital, Ankara, Turkey; 5 Department of Medical Oncology, Okmeydanı Prof. Dr. Cemil Taşcıoğlu City Hospital, Istanbul, Turkey; 6 Department of Medical Oncology, VM Medical Park Hospital, Samsun, Turkey; 7 Department of Medical Oncology, Antalya Training and Research Hospital, Antalya, Turkey; 8 Department of Medical Oncology, Istanbul University Instıtue of Oncology, Istanbul, Turkey; 9 Department of Medical Oncology, Acıbadem Hospital, Kayseri, Turkey; 10 Department of Medical Oncology, Akdeniz University, Antalya, Turkey; 11 Department of Medical Oncology, Zonguldak Hospital, Zonguldak, Turkey; 12 Department of Medical Oncology, Çukurova University, Adana, Turkey; 13 Department of Medical Oncology, Marmara University Pendik Training and Research Hospital, Istanbul, Turkey; 14 Department of Medical Oncology, Adnan Menderes University, Aydın, Turkey; 15 Department of Medical Oncology, Ankara City Hospital, Ankara, Turkey; 16 Department of Medical Oncology, Trakya University, Edirne, Turkey; 17 Department of Medical Oncology, Istanbul Medipol University Bahçelievler Hospital, Istanbul, Turkey; 18 Department of Medical Oncology, Muğla Sıtkı Koçman University, Muğla, Turkey; 19 Mersin Medical Park Hospital, Department of Medical Oncology, Mersin, Turkey; 20 Department of Medical Oncology, Necmettin Erbakan University, Konya, Turkey; 21 Department of Medical Oncology, Celal Bayar University, Manisa, Turkey; 22 Department of Medical Oncology, Gülhane Training and Research Hospital, Ankara, Turkey; 23 Department of Medical Oncology, Afyonkarahisar Health Sciences University Hospital, Afyonkarahisar, Turkey; 24 Department of Medical Oncology, Ankara Medical Park Hospital, Ankara, Turkey; 25 Department of Medical Oncology, Acıbadem University Maslak Hospital, Istanbul, Turkey; 26 Department of Medical Oncology, Hitit University Hospital, Çorum, Turkey; 27 Department of Medical Oncology, Süleyman Demirel University Hospital, Isparta, Turkey; 28 Department of Medical Oncology, Kahramanmaraş Necip Fazıl City Hospital, Kahramanmaraş, Turkey; 29 Department of Medical Oncology, Ersin Arslan Training and Research Hospital, Gaziantep, Turkey; 30 Department of Medical Oncology, UHS Dışkapı Yıldırım Beyazıt Training and Research Hospital, Ankara, Turkey; 31 Department of Medical Oncology, Hacettepe University Instıtue of Oncology, Ankara, Turkey; 32 Department of Medical Oncology, Ankara University, Ankara, Turkey; 33 Department of Medical Oncology, Dicle University, Diyarbakır, Turkey; 34 Department of Medical Oncology, Fırat University, Elazığ, Turkey; 35 Batman Training and Research Hospital, Batman, Turkey; 36 Department of Medical Oncology, Erzincan Binali Yıldırım University, Erzincan, Turkey; 37 Department of Medical Oncology, Atatürk Pulmonary Diseases Hospital, Ankara, Turkey; 38 Department of Medical Oncology, Manisa City Hospital, Manisa, Turkey; 39 Department of Medical Oncology, Gaziantep University, Gaziantep, Turkey; 40 Department of Medical Oncology, Pamukkale University, Denizli, Turkey; 41 Department of Medical Oncology, Namık Kemal University, Tekirdağ, Turkey; 42 Department of Medical Oncology, Ege University, İzmir, Turkey; 43 UHS İzmir Tepecik Training and Research Hospital, İzmir, Turkey; 44 Department of Medical Oncology, Ümraniye Training and Research Hospital, Istanbul, Turkey; 45 Kocaeli Medical Park, Department of Medical Oncology, Kocaeli, Turkey; 46 Department of Medical Oncology, Bahçelievler Memorial Hospital, Istanbul, Turkey; 47 Department of Medical Oncology, Istanbul Medeniyet University, Istanbul, Turkey; 48 Department of Medical Oncology, Meddem Hospital, Isparta, Turkey; 49 Department of Medical Oncology, Kocaeli University, Kocaeli, Turkey; 50 İzmir Bozyaka Training and Research Hospital, İzmir, Turkey; 51 Department of Medical Oncology, Koç University, Istanbul, Turkey; 52 Department of Medical Oncology, American Hospital, Istanbul, Turkey; 53 Department of Medical Oncology, Mustafa Kemal University, Hatay, Turkey

**Keywords:** Advanced breast cancer, Cyclin-dependent kinase, Ribociclib, Palbociclib, Everolimus, Fulvestrant, Endocrine treatment, Hormonotherapy

## Abstract

**Background:**

There is no standard treatment recommended at category 1 level in international guidelines for subsequent therapy after cyclin-dependent kinase 4/6 inhibitor (CDK4/6) based therapy. We aimed to evaluate which subsequent treatment oncologists prefer in patients with disease progression under CDKi. In addition, we aimed to show the effectiveness of systemic treatments after CDKi and whether there is a survival difference between hormonal treatments (monotherapy vs. mTOR-based).

**Methods:**

A total of 609 patients from 53 centers were included in the study. Progression-free-survivals (PFS) of subsequent treatments (chemotherapy (CT, n:434) or endocrine therapy (ET, *n*:175)) after CDKi were calculated. Patients were evaluated in three groups as those who received CDKi in first-line (group A, *n*:202), second-line (group B, *n*: 153) and ≥ 3rd-line (group C, *n*: 254). PFS was compared according to the use of ET and CT. In addition, ET was compared as monotherapy versus everolimus-based combination therapy.

**Results:**

The median duration of CDKi in the ET arms of Group A, B, and C was 17.0, 11.0, and 8.5 months in respectively; it was 9.0, 7.0, and 5.0 months in the CT arm. Median PFS after CDKi was 9.5 (5.0–14.0) months in the ET arm of group A, and 5.3 (3.9–6.8) months in the CT arm (*p* = 0.073). It was 6.7 (5.8–7.7) months in the ET arm of group B, and 5.7 (4.6–6.7) months in the CT arm (*p* = 0.311). It was 5.3 (2.5–8.0) months in the ET arm of group C and 4.0 (3.5–4.6) months in the CT arm (*p* = 0.434). Patients who received ET after CDKi were compared as those who received everolimus-based combination therapy versus those who received monotherapy ET: the median PFS in group A, B, and C was 11.0 vs. 5.9 (*p* = 0.047), 6.7 vs. 5.0 (*p* = 0.164), 6.7 vs. 3.9 (*p* = 0.763) months.

**Conclusion:**

Physicians preferred CT rather than ET in patients with early progression under CDKi. It has been shown that subsequent ET after CDKi can be as effective as CT. It was also observed that better PFS could be achieved with the subsequent everolimus-based treatments after first-line CDKi compared to monotherapy ET.

**Supplementary Information:**

The online version contains supplementary material available at 10.1186/s12885-023-10609-8.

## Background

Approximately 70% of breast cancers are hormone receptor (HR) positive [1]. Endocrine-based treatments are recommended in advanced HR-positive, human epidermal growth factor receptor 2 (Her2)-negative breast cancer without visceral crisis [2, 3]. Progression-free survival (PFS) with monotherapy endocrine treatments was 10–14 months due to endocrine resistance [4]. One of the causes of endocrine resistance was the cyclin-dependent kinase 4/6 (CDK4/6) pathway [4]. A significant PFS contribution of CDK inhibitors has been demonstrated in randomized clinical trials in which CDK4/6 inhibitors were used with endocrine therapies [5, 6]. With the results of these studies, the combination of CDK4/6 inhibitor (CDKi) and endocrine therapy has become the standard of care (SOC) in first-line and second-line therapy [2, 3]. Randomized clinical trials are still underway on which subsequent treatments will be used in patients with progressive disease under CDKi + endocrine therapy. The approximately 7-month progression-free survival obtained in phase 2 ByLieve study, which evaluated the efficacy of alpelisib in patients who had previously received CDKi-based therapy, indicated that alpelisib + fulvestrant might be effective in PIK3CA mutant patients [7]. For patients with progression under CDKi + endocrine therapy, there is currently no standard treatment recommended at category 1 level in international guidelines for subsequent therapy [3]. It is suggested that monotherapy endocrine treatments (fulvestrant or exemestane) or combinations with mTOR inhibitors can be preferred unless there is a visceral crisis. It is also stated that the alpelisib + fulvestrant combination is an option for patients with PIK3CA mutations [3].

In some retrospective studies, it has been observed that physicians prefer chemotherapy after CDKi treatment, even if there is no visceral crisis. In these studies, there was no significant PFS difference between chemotherapy and endocrine therapy. In this multicenter study, we aimed to evaluate which subsequent treatment oncologists prefer in patients with disease progression under CDKi. In addition, we aimed to show the effectiveness of systemic treatments after CDKi and whether there is a survival difference between hormonal treatments (monotherapy vs. mTOR-based).

## Methods

This retrospective study was approved by local ethics committee. Fifty-three centers approved data submission for the study.

Patients with breast cancer aged 18 years or older and with estrogen or progesterone receptor levels ≥ 10% (CDK 4/6 inhibitors were reimbursed for only patients whose tumors expressed ≥ 10% estrogen receptor in our country) who have progressed after CDKi-based therapy and have received at least one systemic therapy (chemotherapy or endocrine-based therapy) were included in the study (between June 2018 and March 2022). Those who received CDKi treatment in early-stage disease and those with Her2 receptor positivity were excluded. Median PFS of the subsequent treatments after CDKi was the primary endpoint. Evaluation of the PFS difference between chemotherapy and endocrine-based treatments was the secondary endpoint.

Patients' age, menopausal status, date of diagnosis and date of metastasis, ECOG performance status, sites of metastasis, median duration of CDKi, treatments they received after CDKi, and dates of progression under treatment were recorded retrospectively from patient files or the hospital registry system. A total of 609 patients included in the study were evaluated in three groups: those who received CDKi on the first line (group A, n:202), those who received it on the second line (group B, n: 153), and those who received it on the ≥ 3rd line ( group C, n:254). Groups A, B, and C were also divided into those who received endocrine therapy (ET) and those who received chemotherapy (CT). The median PFS of the ET and CT groups were compared. In addition, the median PFS of ET was compared in all groups (A, B, C) as monotherapy versus everolimus-based combination therapy.

### Statistical analysis

Continuous variables were presented as median (range or interquartile range (IQR)), and categorical variables as frequency (percent). The Mann–Whitney-U test was used to compare the continuous variables of the two groups, and the chi-square or Fisher's Exact test was used to compare the categorical variables. The time from the start of the subsequent treatment after CDKi to disease progression or death was determined as PFS. Median follow-up time and PFS were determined by the Kaplan–Meier method. The log-rank test was used to determine the median PFS difference between the groups. All statistical analyzes were performed in two ways, and *p* < 0.05 was considered statistically significant.

## Results

### Clinical features of patients at the onset of CDKi

The median age of patients in Groups A, B, and C was 54, 54, and 53, respectively, and the rates of patients ≥ 65 years were 21.3%, 20.3%, and 15.4%. The rates of patients with ECOG PS ≥ 2 were 6.4%, 4.0%, and 5.9% in Groups A, B, and C, respectively. The rates of bone-only metastatic patients in Groups A, B, and C were 36.1%, 37.3%, and 23.6%. The central nervous system (CNS) metastasis rate was 3.5%, 2.0%, and 5.5% in Groups A, B, and C, respectively (Table 1).

**Table 1:** Clinical features of patients before and after CDKi according to their CDKi treatment lines

	CDKi in first line (Group A) *n*:202	CDKi in second line (Group B) *n*:153	CDKi ≥ 3rd line (Group C) *n*:254
**Patient characteristics before CDKi**			
**Age, median (range)**	54 (27–84)	54 (22–87)	53 (26–85)
**Age group year,** ***n*** **(%)**			
< 65	159 (78.7)	122 (79.7)	215 (84.6)
≥ 65	43 (21.3)	31 (20.3)	39 (15.4)
**De-novo metastatic,** ***n*** **(%)**	94 (46.5)	61 (39.9)	109 (42.9)
**Disease-free interval after (neo)adjuvant ET,** ***n*** **(%)**	108 (53.5)	92 (60.1)	145 (57.1)
≤ 24 months	35 (17.3)	25 (16.3)	32 (12.6)
> 24 months	68 (33.7)	55 (35.9)	91 (35.8)
Unknown	5 (2.5)	12 (7.8)	22 (8.7)
**ECOG PS,** ***n*** **(%)**			
0	98 (48.5)	66 (43.1)	96 (37.8)
1	85 (42.1)	77 (50.3)	130 (51.2)
≥ 2	13 (6.4)	6 (4.0)	15 (5.9)
Unknown	6 (3.0)	4 (2.6)	13 (5.1)
**Metastasis site,** ***n*** **(%)**			
Bone only	73 (36.1)	57 (37.3)	60 (23.6)
Bone + lymph node	10 (5.0)	12 (7.8)	9 (3.5)
Visceral only	29 (14.4)	31 (20.3)	43 (16.9)
Bone + visceral	90 (44.6)	53 (34.6)	142 (55.9)
**Visceral metastasis site,** ***n*** **(%)**			
CNS	7 (3.5)	3 (2.0)	14 (5.5)
Liver	43 (21.3)	44 (28.8)	110 (43.4)
Lung	59 (29.2)	45 (29.4)	93 (36.6)
**Patient characteristics before subsequent treatment after CDKi**			
**Median duration of CDKi, months (range)**	10 (2–46)	9 (2–34)	5 (2–24)
ET	17 (3–46)	11 (3–34)	8.5 (3–23)
CT	9 (2–39)	7 (2–20)	5 (2–24)
**Duration of CDKi,** *n* **(%)**			
< 6 months	42 (20.8)	44 (28.8)	131 (51.6)
≥ 6 months	160(79.2)	109 (71.2)	123 (48.4)
**Age, median (range)**	55 (28–86)	55 (23–87)	54 (26–86)
**Age group year,** ***n*** **(%)**			
< 65	150 (74.3)	121 (79.1)	209 (82.3)
≥ 65	52 (25.7)	32 (20.9)	45 (17.7)
**ECOG PS,** ***n*** **(%)**			
0	91 (45.0)	64 (41.8)	94 (37.0)
1	98 (48.5)	82 (53.6)	142 (55.9)
≥ 2	13 (6.4)	7 (4.6)	18 (7.1)
**Metastasis site,** ***n*** **(%)**			
Bone only	46 (22.8)	32 (20.9)	32 (12.6)
Bone + lymph node	8 (4.0)	8 (5.2)	7 (2.8)
Visceral only	21 (10.4)	18 (11.8)	32 (12.6)
Bone + visceral	127 (62.9)	95 (62.1)	183 (72.0)

*CDKi* Cyclin dependent kinase inhibitor, *ET* Endocrine therapy, *CT* Chemotherapy, *CNS* Central nervous system, *ECOG PS* Eastern Cooperative Oncology Group Performance Status

### Clinical features of patients after CDKi

The median duration of CDKi in Group A was 10 months (range: 3–46). In group A, median CDKi was 17 months (range: 3–46 months) in the ET arm and 9 months (range: 2–39 months) in the CT arm. The median duration of CDKi in Group B was 9 months (range: 2–34). In group B, median CDKi was 11 months (range: 3–34 months) in the ET arm and 7 months (range: 2–20 months) in the CT arm. The median duration of CDKi in Group C was 5 months (range: 2–24). In group C, median CDKi was 8.5 months (range: 3–23 months) in the ET arm and 5 months (range: 2–24 months) in the CT arm. The rate of bone-only metastatic patients was 22.8%, 20.9%, and 12.6% in groups A, B, and C, respectively (Table 1).

### Subsequent treatments after CDKi

**Fig. 1:**
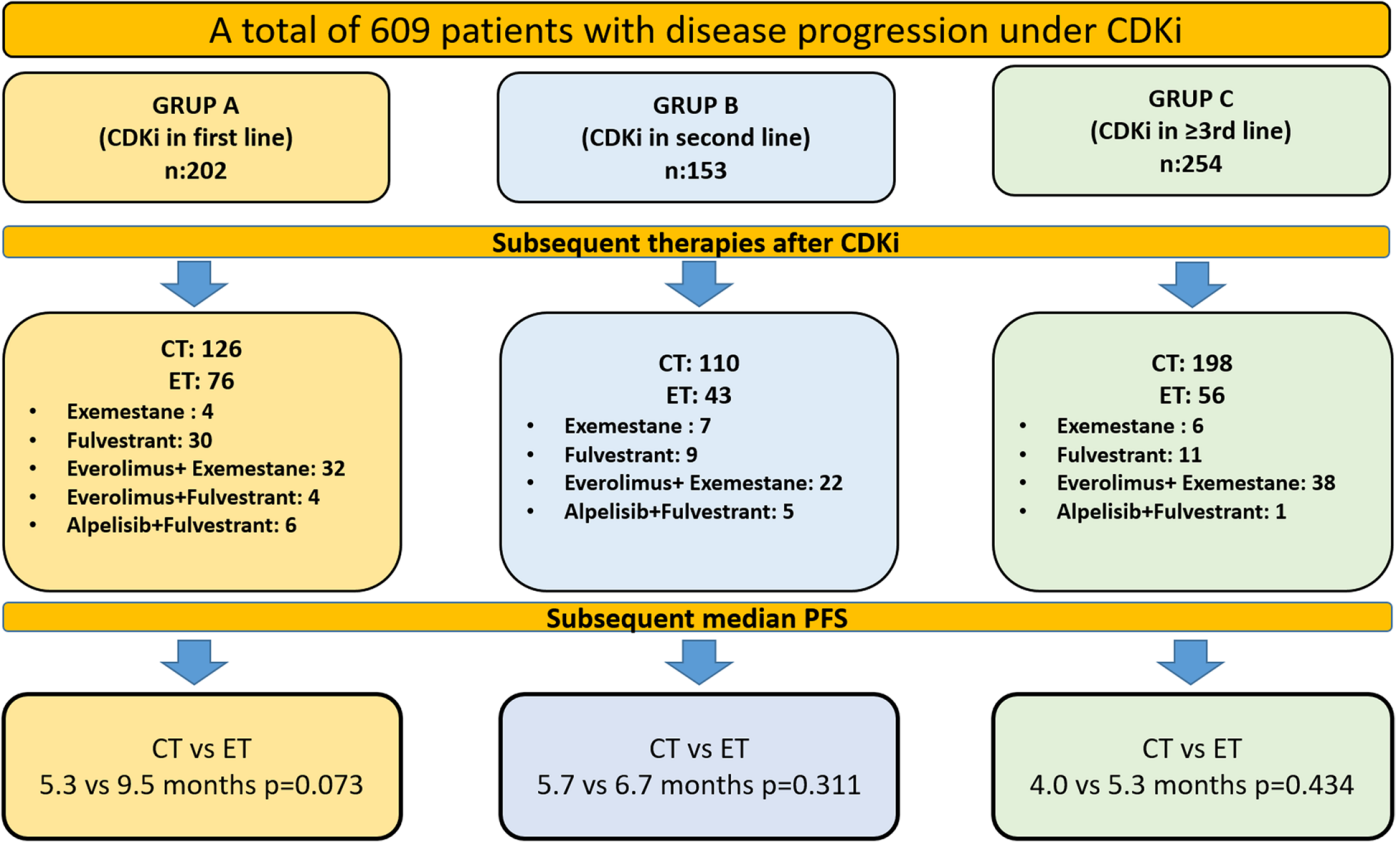
Subsequent therapies and median progression free survivals of the patients

In Group A after CDKi, 126 (62.4%) patients received CT, 76 (37.6%) ET; 110 (71.9%) CT, 43 (28.1%) ET in Group B; in Group C, 198 (77.9%) received CT and 56 (22.1%) ET (Fig. 1). The most frequently used chemotherapies in all three groups were capecitabine and taxane (Supp Table 1). Of the patients in group A who received ET, 4 received exemestane, 30 received fulvestrant, 32 received everolimus + exemestane, 4 received everolimus + fulvestrant, and 6 received Alpelisib + fulvestrant. In group B, 7 patients received exemestane, 9 received fulvestrant, 22 received everolimus + exemestane, and 5 received alpelisib + fulvestrant. In group C, 6 patients received exemestane, 11 received fulvestrant, 38 received everolimus + exemestane, and 1 received alpelisib + fulvestrant (Fig. 1).

### Survival outcomes

Median follow-up was 6.2 months (95% CI: 4.6–7.9 months) in Group A, 7.5 months (95% CI: 5.7–9.5 months) in the ET arm, and 5.1 months (95% CI: 4.4–5.8 months) in the CT arm of group A. Median follow-up was 6.5 months (95% CI: 5.0–7.9 months) in Group B, 7.9 months (95% CI: 5.8–9.9 months) in the ET arm, and 5.3 months (95% CI: 4.7–5.9 months) in the CT arm of group B. Median follow-up was 7.5 months (95% CI: 6.7–8.4 months) in Group C, 7.6 months (95% CI: 6.2–8.9 months) in the ET arm, and 6.9 months (95% CI: 3.9–9.9 months) in the CT arm of group C.

The subsequent median PFS after CDKi was 9.5 (5.0–14.0) months in the ET arm and 5.3 (3.9–6.8) months in the CT arm (*p* = 0.073) of group A. Median PFS was 6.7 (5.8–7.7) months in the ET arm and 5.7 (4.6–6.7) months in the CT arm (*p* = 0.311) of group B. Median PFS was 5.3 (2.5–8.0) months in the ET arm and 4.0 (3.5–4.6) months in the CT arm (*p* = 0.434) of group C (Fig. 1).

### Clinical characteristics and survival outcomes of monotherapy and everolimus-based treatment groups

In Groups A, B, and C, the median duration of CDKi, median age, ECOG PS, and metastasis sites were similar in monotherapy and everolimus-based arms. The rate of denovo metastatic patients in the monotherapy arm of Group A was higher than in the everolimus-based arm (63.6% vs. 36.1%, *p* = 0.022).

**Fig. 2:**
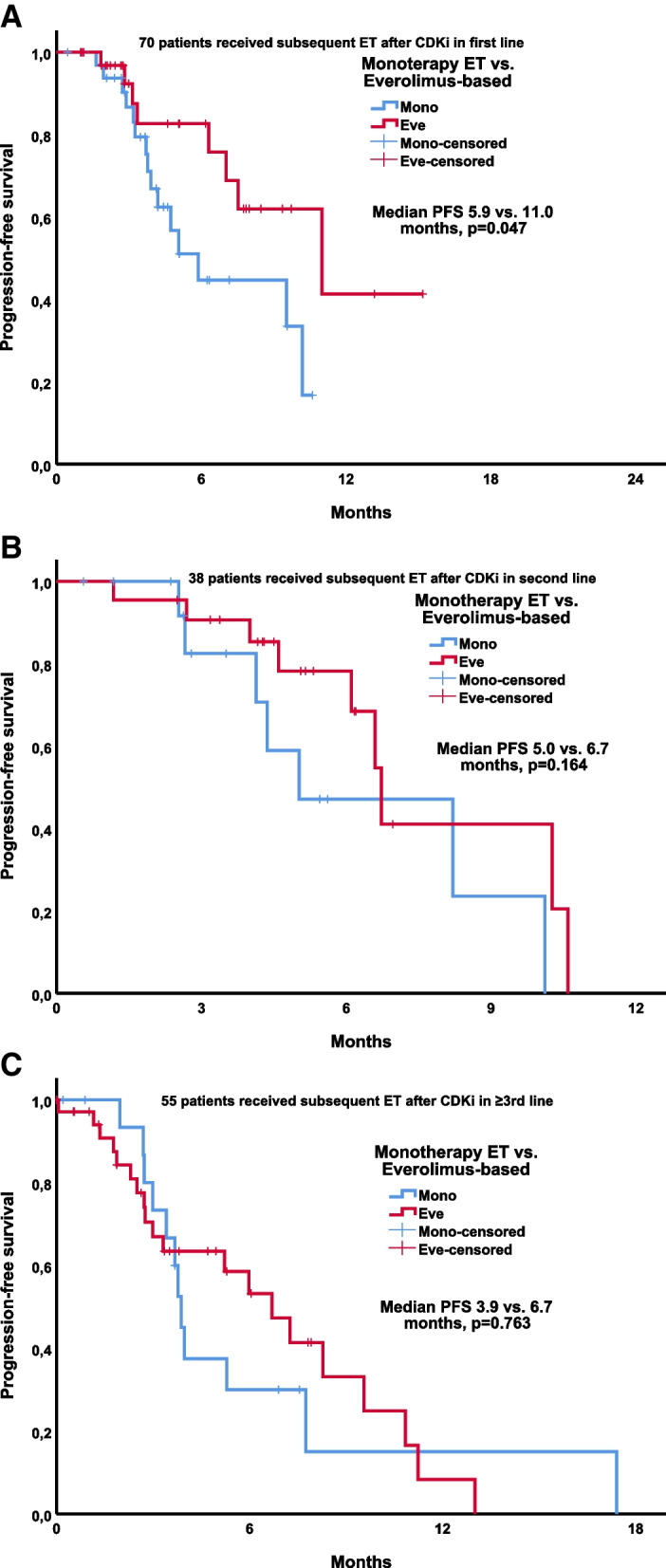
**A**. Progression free survival according to endocrine treatment in patients who take CDKi in first line. **B**. Progression free survival according to endocrine treatment in patients who take CDKi in second line. **C**. Progression free survival according to endocrine treatment in patients who take CDKi in ≥ 3rd line

In Group A, the rate of patients who received ET in the adjuvant setting and relapsed in the first 24 months was higher in the monotherapy arm than in the everolimus-based arm (46.2% vs. 26.1%, *p* = 0.044) (Table 2). When patients who received ET after CDKi were compared as those who received everolimus-based combination therapy versus those who received monotherapy ET, the median PFS of everolimus-based and monotherapy arms in groups A, B, and C was 11.0 vs. 5.9 (*p* = 0.047) months, 6.7 vs. 5.0 (*p* = 0.164) months, and 6.7 vs. 3.9 (*p* = 0.763) months, respectively (Fig. 2A-C).

**Table 2:** Comparison of clinical features of patients receiving monotherapy ET and everolimus-based therapy

	Group A (CDKi in first line)			Group B (CDKi in second line)			Group C (CDKi in ≥ 3rd line)		
	Monotherapy ET *n*:34	Everolimus-based therapy *n*:36	*p*-value	Monotherapy ET *n*:16	Everolimus-based therapy *n*:22	*p*-value	Monotherapy ET *n*:17	Everolimus-based therapy *n*:38	*p*-value
**Median duration of CDKi, months (range)**	15 (9–46)	19 (3–31)	0.410	10 (3–34)	13 (5–26)	0.126	7 (3–14)	11 (3–23)	0.058
**Age, median (range)**	57 (31–81)	57 (37–86)	0.920	53 (23–75)	59 (37–74)	0.293	58 (31–81)	53 (36–80)	0.392
**ECOG PS,** ***n*** **(%)**									
0	16 (47.1)	15 (41.7)	0.650	7 (43.8)	12 (54.5)	0.511	3 (17.6)	17 (44.7)	0.054
≥ 1	18 (52.9)	21 (58.3)		9 (56.3)	10 (45.5)		14 (82.4)	21 (55.3)	
**De-novo metastatic,** ***n*** **(%)**	21 (63.6)	13 (36.1)	0.022	6 (46.2)	8 (36.4)	0.568	7 (43.8)	16 (44.4)	0.963
**Disease-free interval after (neo)adjuvant ET,** ***n*** **(%)**									
≤ 24 months	6 (46.2)	6 (26.1)	0.044	1 (10.0)	3 (21.4)	0.558	5 (50.0)	12 (54.5)	0.321
> 24 months	5 (38.5)	17 (73.9)		6 (60.0)	9 (64.3)		4 (40.0)	4 (18.2)	
Unknown	2 (15.4)	0 (0)		3 (30.0)	2 (14.3)		1 (10.0)	6 (27.3)	
**Post-CDKi metastatic site,** ***n*** **(%)**									
Bone only	16 (47.1)	13 (36.1)	0.175	6 (37.5)	5 (22.7)	0.288	0 (0)	12 (31.6)	0.061
Bone + lymph node	3 (8.8)	1 (2.8)		2 (12.5)	1 (4.5)		1 (5.9)	1 (2.6)	
Visceral only	1 (2.9)	6 (16.7)		2 (12.5)	1 (4.5)		3 (17.6)	3 (7.9)	
Bone + visceral	14 (41.2)	16 (44.4)		6 (37.5)	15 (68.2)		13 (76.5)	22 (57.9)	

*CDKi* Cyclin dependent kinase inhibitor, *ET* Endocrine therapy, *ECOG PS* Eastern Cooperative Oncology Group Performance Status

Univariate PFS analysis of patients who received endocrine therapy after CDKi in the first line (*n*:70) was shown in Table 3. Age, ECOG PS, the median duration of CDKi, denovo metastasis, metastatic site, and disease-free interval did not affect PFS.

**Table 3:** Univariate PFS analysis of patients received endocrine therapy after CDKi in first line (*n*:70)

Age (year)		
< 65	7.5 (4.3–10.7)	0.359
≥ 65	NR	
**ECOG PS**		
0	7.5 (3.4–11.6)	0.739
≥ 1	9.5 (4.9–15.6)	
**Denovo metastatic disease**		
No	7.5 (4.2–10.8)	0.883
Yes	9.5 (3.0–16.0)	
**Disease-free interval after (neo)adjuvant ET,** ***n*** (%)		
≤ 24 months	5.1 (4.7–10.3)	0.136
> 24 months	NR	
**Duration of CDKi**		
≤ 17 months	10.2 (1.6–18.7)	0.647
> 17 months	9.5 (5.8–13.2)	
**Post-CDKi metastatic site, *n*** **(%)**		
Bone only	9.5 (4.2–14.9)	0.429
Bone + lymph node	NR	
Visceral only	3.8 (0–8.4)	
Bone + visceral	NR	
**Endocrine therapy**		
Monotherapy	5.9 (3.8–7.9)	0.047
Everolimus-based	11.0 4.8 (17.2)	

*CDKi* Cyclin dependent kinase inhibitor, *ET* Endocrine therapy, *ECOG PS* Eastern Cooperative Oncology Group Performance Status, *PFS* Progression-free survival

### Safety data

Everolimus initiation dose was 10 mg/day. Dose reduction (to 5 mg) was performed in 19.1% of the patients. In the everolimus-based group, 42% of the patients had Grade 1 stomatitis, and 11% had Grade 2 stomatitis. There were no data on the use of primary dexamethasone prophylaxis for stomatitis. In the everolimus-based group, 15% of the patients had elevated AST or ALT, and 17% had arthralgia. In the monotherapy ET group, the most common adverse event was arthralgia, with a rate of 15%.

Any grade of adverse events occurred in 93% of patients receiving chemotherapy. 84% of patients who received CT had at least one dose reduction. The most common adverse events were neutropenia (47%), anemia (38%), and fatigue (33%). There was no patient who had discontinued CT due to toxicity.

## Discussion

A standard of care treatment recommended as subsequent therapy in patients with advanced HR + , Her2- breast cancer that has progressed under CDKi therapy has not yet been established. Results of ongoing randomized clinical trials are awaited. Therefore, real-life data of retrospective studies is crucial. In our study, the factors affecting subsequent treatment choices and the effectiveness of these treatments were evaluated. In this multicenter retrospective study, it was observed that the short duration of CDKi in patients with HR + Her2- advanced breast cancer that progressed under CDKi treatment increased physicians' preference for CT in subsequent treatment. There was no difference in PFS between the subsequent CT and ET arms. When endocrine-based treatments were compared as monotherapy vs. everolimus-based treatments among patients who received CDKi in first-line, longer PFS was found with everolimus-based treatments.

In a study evaluating the factors affecting treatment choices (CT vs. ET) after CDKi, priority ET was preferred as subsequent therapy in patients who received CDKi in the first line, and priority CT was preferred in those who received CDKi in the second line [8]. As a result of the multivariate analysis performed in the same study, young age and short duration of CDKi were independent factors predicting CT preference [8]. In our study, physicians preferred CT for subsequent treatment in patients with a short duration of CDKi use.

PALOMA 3, a randomized clinical trial comparing fulvestrant vs. fulvestrant + palbociclib in previously treated patients with advanced breast cancer, showed no difference in duration of treatment between subsequent CT and ET after palbociclib (5.6 vs. 4.3 months) [9]. Similarly, retrospective analyzes of TREND, a phase2 study, showed no difference in duration of treatment between subsequent CT and ET (4.6 vs. 3.7 months), regardless of palbociclib use [10]. A retrospective study evaluating subsequent treatments after palbociclib found no significant difference in subsequent PFS between CT and ET, regardless of the palbociclib line [11]. The number of patients evaluated in Xi et al.'s study was limited [11]. For example, there were seven patients in both CT and ET arms after the first line of palbociclib [11]. The median duration of palbociclib in the first line was 20.7 months, similar to PALOMA3 [11]. The median PFS was 17 months in patients (*n* = 7) who received ET after the first line. The median duration of palbociclib in the second line was 12.8 months. In this setting, the median PFS of subsequent ET (*n* = 9) was 9.3 months, and CT (*n* = 14) was 4.7 months [11]. The subsequent PFS of patients who received palbociclib at the third or more line was 4.2 months in the ET arm (*n* = 16) and 4.1 months in the CT arm (*n* = 49) [11]. Similarly, in our study, the PFS of those who received subsequent CT and ET was 5.3 vs. 9.5, 5.7 vs. 6.7, and 4.0 vs. 5.3 months, respectively, in patients who received first, second, and ≥ 3rd line CDKi, and no statistical difference was found. In our study, short PFS obtained with subsequent treatments after the first line was associated with a short median duration of CDKi. The short use of the median CDKi indicated a relatively poor prognostic patient population in this study.

It was demonstrated in the BOLERO-2 study that the everolimus + exemestane combination achieved longer PFS than monotherapy exemestane [12]. In this study, 54% of the included patients received at least three lines of therapy [12]. The median PFS of the everolimus + exemestane combination was 6.9 months according to the local investigator's evaluation and 10.6 months according to the central investigator's evaluation [12]. At the time of the study, CDKi was not yet in use [12].

Contradictory results were obtained from limited retrospective studies showing the efficacy of everolimus-based treatments after CDKi [13–15]. In the study by Rozenblit et al., the median time to next treatment (TTNT) of those who received everolimus + exemestane who progressed under one line of monotherapy ET was longer than those who had disease progression under CDKi + ET (6.2 vs. 4.4 months, *p* = 0.03) [13]. Another small retrospective study evaluating everolimus-based therapy after palbociclib found a median PFS of 4.2 months [14]. However, 83% of the 41 patients included in this study consisted of patients who received at least three lines of treatment (heavy treatment) [14]. In a retrospective study comparing the efficacy of everolimus + exemestane in CDK-naive (*n* = 26) and CDK-received (*n* = 17) patients, median PFS was 4.2 vs. 3.6 months [15]. The authors suggested that the efficacy of everolimus + exemestane was not affected by CDKi [15]. In the same study, it was also noted that the median duration of CDKi was short (median CDKi duration of 10.3 months) [15]. In our study, among patients who received CDKi in first-line, those who received subsequent everolimus-based therapy had longer PFS than those who received monotherapy ET (11.0 vs. 5.9 months). The data obtained from these studies support that the mTOR/AKT/PI3K pathway, one of the many resistance mechanisms against CDKi, may be a target for subsequent therapies.

Our study had some limitations. The main limitations were that the study was retrospective, and the median duration of CDKi and follow-up were short. More patients received CT in the subsequent treatment than those who received ET. In addition, the shorter median duration of CDKi in patients who received CT compared to ET suggested that this group might have a relatively poor prognosis. The difference in median duration CDKi may have caused bias in the results obtained by comparing the CT and ET groups. The short median duration of CDKi could also affect subsequent PFS. Another limitation was that the rate of patients with disease progression in the first 24 months after adjuvant ET was lower in those receiving everolimus-based therapy than those receiving monotherapy ET. Despite these limitations, the investigation of the efficacy of subsequent treatments after CDKi with a large patient population (*n* = 609) was the strength of our study.

## Conclusion

It was observed that oncologists preferred CT rather than ET in patients whose disease progressed in a short time with CDKi. This study showed that subsequent ET could be as effective as CT in patients whose disease progressed under ET + CDKi treatment. In addition, better PFS could be obtained with the subsequent everolimus-based therapy than with monotherapy ET after first line CDKi.

## Supplementary Information

**Additional file 1: TableS1.** Chemotherapy regimens.

## Data Availability

The database of the study is available in the corresponding author and will be sent when requested by e-mail.

## Abbreviations

CDK: Cyclin-dependent kinase
CDKi: Cyclin dependent kinase inhibitor
CNS: Central nervous system
CT: Chemotherapy
ECOG PS: Eastern Cooperative Oncology Group Performance Status
ET: Endocrine treatment
HR: Hormone receptor
PFS: Progression-free survival
