# Correction: Efficacy of subsequent treatments in patients with hormone-positive advanced breast cancer who had disease progression under CDK 4/6 inhibitor therapy

**DOI:** 10.1186/s12885-023-10662-3

**Published:** 2023-02-27

**Authors:** Cengiz Karacin, Berna Oksuzoglu, Ayşe Demirci, Merve Keskinkılıç, Naziyet Köse Baytemür, Funda Yılmaz, Oğuzhan Selvi, Dilek Erdem, Esin Avşar, Nail Paksoy, Necla Demir, Sema Sezgin Göksu, Sema Türker, Ertuğrul Bayram, Abdüssamet Çelebi, Hatice Yılmaz, Ömer Faruk Kuzu, Seda Kahraman, İvo Gökmen, Abdullah Sakin, Ali Alkan, Erdinç Nayır, Muzafer Uğraklı, Ömer Acar, İsmail Ertürk, Hacer Demir, Ferit Aslan, Özlem Sönmez, Taner Korkmaz, Özde Melisa Celayir, İbrahim Karadağ, Erkan Kayıkçıoğlu, Teoman Şakalar, İlker Nihat Öktem, Tülay Eren, Enes Erul, Eda Eylemer Mocan, Ziya Kalkan, Nilgün Yıldırım, Yakup Ergün, Baran Akagündüz, Serdar Karakaya, Engin Kut, Fatih Teker, Burçin Çakan Demirel, Kubilay Karaboyun, Elvina Almuradova, Olçun Ümit Ünal, Abdilkerim Oyman, Deniz Işık, Kerem Okutur, Buğra Öztosun, Burcu Belen Gülbağcı, Mehmet Emin Kalender, Elif Şahin, Mustafa Seyyar, Özlem Özdemir, Fatih Selçukbiricik, Metin Kanıtez, İsa Dede, Mahmut Gümüş, Erhan Gökmen, Arzu Yaren, Serkan Menekşe, Senar Ebinç, Sercan Aksoy, Gökşen İnanç İmamoğlu, Mustafa Altınbaş, Bülent Çetin, Başak Oyan Uluç, Özlem Er, Nuri Karadurmuş, Atike Pınar Erdoğan, Mehmet Artaç, Özgür Tanrıverdi, İrfan Çiçin, Mehmet Ali Nahit Şendur, Esin Oktay, İbrahim Vedat Bayoğlu, Semra Paydaş, Adnan Aydıner, Derya Kıvrak Salim, Çağlayan Geredeli, Tuğba Yavuzşen, Mutlu Doğan, İlhan Hacıbekiroğlu

**Affiliations:** 1 Department of Medical Oncology, UHS Dr Abdurrahman Yurtaslan Ankara Oncology Training and Research Hospital, Ankara, Turkey; 2 Department of Medical Oncology, Sakarya University, Sakarya, Turkey; 3 Department of Medical Oncology, Dokuz Eylül University, İzmir, Turkey; 4 Department of Medical Oncology, Memorial Hospital, Ankara, Turkey; 5 Department of Medical Oncology, Okmeydanı Prof. Dr. Cemil Taşcıoğlu City Hospital, Istanbul, Turkey; 6 Department of Medical Oncology, VM Medical Park Hospital, Samsun, Turkey; 7 Department of Medical Oncology, Antalya Training and Research Hospital, Antalya, Turkey; 8 Department of Medical Oncology, Istanbul University Instıtue of Oncology, Istanbul, Turkey; 9 Department of Medical Oncology, Acıbadem Hospital, Kayseri, Turkey; 10 Department of Medical Oncology, Akdeniz University, Antalya, Turkey; 11 Department of Medical Oncology, Zonguldak Hospital, Zonguldak, Turkey; 12 Department of Medical Oncology, Çukurova University, Adana, Turkey; 13 Department of Medical Oncology, Marmara University Pendik Training and Research Hospital, Istanbul, Turkey; 14 Department of Medical Oncology, Adnan Menderes University, Aydın, Turkey; 15 Department of Medical Oncology, Ankara City Hospital, Ankara, Turkey; 16 Department of Medical Oncology, Trakya University, Edirne, Turkey; 17 Department of Medical Oncology, Istanbul Medipol University Bahçelievler Hospital, Istanbul, Turkey; 18 Department of Medical Oncology, Muğla Sıtkı Koçman University, Muğla, Turkey; 19 Mersin Medical Park Hospital, Department of Medical Oncology, Mersin, Turkey; 20 Department of Medical Oncology, Necmettin Erbakan University, Konya, Turkey; 21 Department of Medical Oncology, Celal Bayar University, Manisa, Turkey; 22 Department of Medical Oncology, Gülhane Training and Research Hospital, Ankara, Turkey; 23 Department of Medical Oncology, Afyonkarahisar Health Sciences University Hospital, Afyonkarahisar, Turkey; 24 Department of Medical Oncology, Ankara Medical Park Hospital, Ankara, Turkey; 25 Department of Medical Oncology, Acıbadem University Maslak Hospital, Istanbul, Turkey; 26 Department of Medical Oncology, Hitit University Hospital, Çorum, Turkey; 27 Department of Medical Oncology, Süleyman Demirel University Hospital, Isparta, Turkey; 28 Department of Medical Oncology, Kahramanmaraş Necip Fazıl City Hospital, Kahramanmaraş, Turkey; 29 Department of Medical Oncology, Ersin Arslan Training and Research Hospital, Gaziantep, Turkey; 30 Department of Medical Oncology, UHS Dışkapı Yıldırım Beyazıt Training and Research Hospital, Ankara, Turkey; 31 Department of Medical Oncology, Hacettepe University Instıtue of Oncology, Ankara, Turkey; 32 Department of Medical Oncology, Ankara University, Ankara, Turkey; 33 Department of Medical Oncology, Dicle University, Diyarbakır, Turkey; 34 Department of Medical Oncology, Fırat University, Elazığ, Turkey; 35 Batman Training and Research Hospital, Batman, Turkey; 36 Department of Medical Oncology, Erzincan Binali Yıldırım University, Erzincan, Turkey; 37 Department of Medical Oncology, Atatürk Pulmonary Diseases Hospital, Ankara, Turkey; 38 Department of Medical Oncology, Manisa City Hospital, Manisa, Turkey; 39 Department of Medical Oncology, Gaziantep University, Gaziantep, Turkey; 40 Department of Medical Oncology, Pamukkale University, Denizli, Turkey; 41 Department of Medical Oncology, Namık Kemal University, Tekirdağ, Turkey; 42 Department of Medical Oncology, Ege University, İzmir, Turkey; 43 UHS İzmir Tepecik Training and Research Hospital, İzmir, Turkey; 44 Department of Medical Oncology, Ümraniye Training and Research Hospital, Istanbul, Turkey; 45 Kocaeli Medical Park, Department of Medical Oncology, Kocaeli, Turkey; 46 Department of Medical Oncology, Bahçelievler Memorial Hospital, Istanbul, Turkey; 47 Department of Medical Oncology, Istanbul Medeniyet University, Istanbul, Turkey; 48 Department of Medical Oncology, Meddem Hospital, Isparta, Turkey; 49 Department of Medical Oncology, Kocaeli University, Kocaeli, Turkey; 50 İzmir Bozyaka Training and Research Hospital, İzmir, Turkey; 51 Department of Medical Oncology, Koç University, Istanbul, Turkey; 52 Department of Medical Oncology, American Hospital, Istanbul, Turkey; 53 Department of Medical Oncology, Mustafa Kemal University, Hatay, Turkey

**Correction: BMC Cancer 23, 136 (2023)**

**https://doi.org/10.1186/s12885-023-10609-8**

Following publication of the original article [1], the authors reported an error in the author name of Enes Erul.

Incorrect: Enes Urul

Correct: Enes Erul

The original article [1] has been corrected.
