# Supplementary material for: Efficacy of subsequent treatments in patients with hormone-positive advanced breast cancer who had disease progression under CDK 4/6 inhibitor therapy
Source: BMC Cancer. 2023 Feb 10;23:136. doi: 10.1186/s12885-023-10609-8 (PMC9912535; doi:10.1186/s12885-023-10609-8)
Supplement: Supplementary file 1 — Additional file 1: TableS1. Chemotherapy regimens. [file 12885_2023_10609_MOESM1_ESM.docx]

| Supplemental Table S1. Chemotherapy regimens | | | |
| --- | --- | --- | --- |
| Taxane | 48 | 32 | 43 |
| Capecitabine | 37 | 44 | 50 |
| Carboplatin + Taxane | 17 | 9 | 23 |
| Anthracycline + cyclophosphamide | 12 | 4 | 6 |
| Gemcitabine | 5 | 7 | 24 |
| Cisplatin + gemcitabine | 4 | 10 | 15 |
| Taxane + cyclophosphamide | 3 | 0 | 0 |
| Vinorelbine | 0 | 4 | 22 |
| Eribuline | 0 | 0 | 10 |
| Ixabepilone | 0 | 0 | 3 |
